# Medical students’ views on what professionalism means: an Ubuntu perspective

**DOI:** 10.1007/s10459-023-10280-5

**Published:** 2023-09-15

**Authors:** Mantoa Mokhachane, Lionel Green-Thompson, Ann George, Tasha Wyatt, Ayelet Kuper

**Affiliations:** 1 University of Witwatersrand, Johannesburg, South Africa; 2 University of Cape Town, Cape Town, South Africa; 3 Uniformed University of the Health Sciences, Bethesda, USA; 4 University of Toronto, Toronto, Canada

**Keywords:** Professionalism, Medical clerkship experiences, Ubuntu, Physicians Charter, South Africa

## Abstract

Medical training has become a global phenomenon, and the Physician’s Charter (PC), as a missionary document, is key to training those outside the Global North. Undergraduate and postgraduate students in the medical profession are sometimes trained in contexts foreign to their social and ontological backgrounds. This might lead to confusion and blunders, creating an impression of what might look and feel unprofessional to those unfamiliar with the local context. Understanding the cultural backgrounds of the trainees is crucial, and the reverse is also as important. It is essential for clinicians and trainees to understand the cultural backgrounds of their patients to avoid miscommunication. In this phenomenological study, we recruited participants in 2020 who were in their first to fourth year of study of medical training during the #FeesMustFall protests. We used data from this extensive study looking at students’ experiences during their training amidst protest and social upheavals in a South African tertiary institution. For this paper, we examined what professionalism means to the student participants using an African Ubuntu lens. Ubuntu and the Collective Finger theory were used to investigate what professionalism means to participants. The Ubuntu philosophy was compared to the PC. In the findings, the clinical space is hierarchical, silencing and the opposite of what Ubuntu means. In comparison to the PC, respect is overarching while compassion and responsibility are the most comparable to the Charter. This study adds an African voice to the professionalism discourse while showing African elements that could be aligned to the PC to challenge the current global discourses.

## Introduction

The Physician's Charter has been invoked as a way of globalizing professionalism; however, it has been influenced only by voices situated in the Global North and Europe (Olive & Abercrombie, 2017). In an attempt to assess its relevance elsewhere, ideas around professionalism have been examined through other cultural lenses, demonstrating the ways in which this construct is both aligned and misaligned with other cultures. These studies are important in that they show that the Physician’s Charter and professionalism, more generally, is in fact culturally bound. However, until now research has yet to compare the culture of Ubuntu within South Africa with these dominant ideas surrounding professionalism. This is problematic because many Southern African physicians work and train in Europe and the Global North and bring their cultural upbringing and training with them. And research shows that there is often cultural misunderstandings around professionalism when physicians from outside of North America work in this setting (Gosselin et al., 2016).

Given that our research team has consistently heard scholars from outside Africa discussing professionalism and the Physician’s Charter in their contexts, and no published literature originates from Africa or Southern Africa, our work focuses on this topic. In this study, we add to the voices outside of North America and Europe by focusing on professionalism from Southern Africa using a local, ontological perspective known as Ubuntu, the connecting thread in Southern Africa (Mokhachane et al., 2022). We are studying Ubuntu and professionalism in Southern Africa because not only is there is a paucity of data on this topic (Mokhachane et al., 2022), but it is important to understand the relevance and fit of outside constructs within local contexts.

### Physician’s Charter and professionalism across the globe

The Physician’s Charter has three fixed fundamental principles: the primacy of patient welfare—the patient should be placed above that of the physician, autonomy—respecting patient’s autonomy, and social justice—promotion of social justice in the healthcare system (Blank, 2002). The Charter also added a commitment to a set of professional responsibilities such as professional competence, honesty with patients, patient confidentiality, scientific knowledge, improving quality of care, improving access to care, a just distribution of finite resources and scientific knowledge (Blank, 2002; Blank et al., 2003). Over the years, scholars in several countries have compared what is stipulated in the Charter to their local settings and different contexts to elicit its applicability (Bano et al., 2021; Ho et al., 2016; Ibrahim et al., 2021; Jin, 2015; Nishigori et al., 2014; Van Rooyen et al., 2004; Van Rooyen & Treadwell, 2007). Some scholars have contrasted the Western notion of professionalism or the Physician’s Charter to other ways of knowing (Al-Eraky et al., 2014; Jotkowitz & Glick, 2005; Nishigori et al., 2014). Nishigori contrasted the Physician’s Charter to Bushido (a Japanese code of conduct based on the ancient samurai warriors), while Jotkowitz compared it to the Jewish ethical perspective (Jotkowitz & Glick, 2005; Nishigori et al., 2014). Nishigori found similarities when comparing the Japanese concept of Bushido to the Physician’s Charter (Nishigori et al., 2014). On the other hand, Jotkowitz mentions that the primacy of the patient in the Physician’s Charter concords with Jewish tradition (Jotkowitz & Glick, 2005). However, he goes on to say that this first principle of primacy of the patient has been challenged as contradicting social justice (Jotkowitz & Glick, 2005; Reiser & Banner, 2003). Although Jin and Ho found similarities between the Physician’s Charter and the Chinese and Arabic charters, they also found differences or divergences regarding autonomy which is replaced or dominated by family-centered decision-making (Ho et al., 2016; Jin, 2015). Writing from Pakistan, Bano expressed concerns that the Physicians Charter did not consider patients’ views (Bano et al., 2021). In combination, all of these studies demonstrate that although there are some similarities in the Physicians Charter and other cultural contexts, there are also differences that cannot be ignored. It is therefore pertinent for those outside the Global North interrogate this in their setting.

Other scholars, while not dealing specifically with the Physician’s Charter, contrasted the Western notion of professionalism to their cultural or religious context (Al-Eraky et al., 2014; Ho et al., 2011). For example, Al-Eraky looked at the faith-rooted Arabian Four Gates model of Medical professionalism “grounded in the mind of the medical professional” (Al-Eraky et al., 2014). In the Four Gates model, the inner gate deals with the self, while the overarching gate deals with God. The second innermost gate deals with the task, and the third deals with others (Al-Eraky et al., 2014). Ho and colleagues questioned the appropriateness of the Western framework of medical professionalism and suggested that a professionalism framework should be culturally relevant to the local context (Ho et al., 2011). She explored tensions between western medicine and Taiwanese culture, identifying intercultural dilemmas where the western notion of professionalism was at odds with local values (Ho et al., 2017). The centrality of self-integrity, integrating the professional and personal roles, was the core difference between Taiwanese and Western notions of professionalism (Ho et al., 2011), with Taiwanese frameworks displaying morality and integrity as dominant features, possibly informed by Confucianism (Ho et al., 2014). In some countries, including South Africa, researchers found that students disagreed with some aspects of the Physician’s Charter (Ding et al., 2018; van Rooyen, 2004; van Rooyen & Treadwell, 2007). South African students stressed the humanistic aspects of professionalism lacking in the Physician’s Charter, while Chinese students endorsed the Physicians Charter (Ding et al., 2018; van Rooyen & Treadwell, 2007). These studies confirm that cultural and religious backgrounds play a role in shaping an individual’s professionalism in their settings.

These contradictions are not surprising, as every culture has a different way of looking at professionalism and what it means in their cultural or religious context. (Ho et al., 2014; Nie et al., 2015; Nishigori et al., 2014). These are all based on different ontological systems, which include the European post-enlightenment antecedents of the Physicians Charter, notions from Asian countries such as China, Taiwan and Japan, ways of thinking and knowing from the Middle East, and others. However, there is extraordinarily little documentation on what professionalism means in Africa, especially Southern Africa, particularly without the overlay of European influences. Africa is a large, heterogenous continent, and conceptions of professionalism differ from region to region based on the dominant ontological system. However, in Southern Africa, Ubuntu (or its cognates in different languages) is the ontological thread that connects the vastly different cultures across the region.

### Professionalism and Ubuntu

**Fig. 1:**
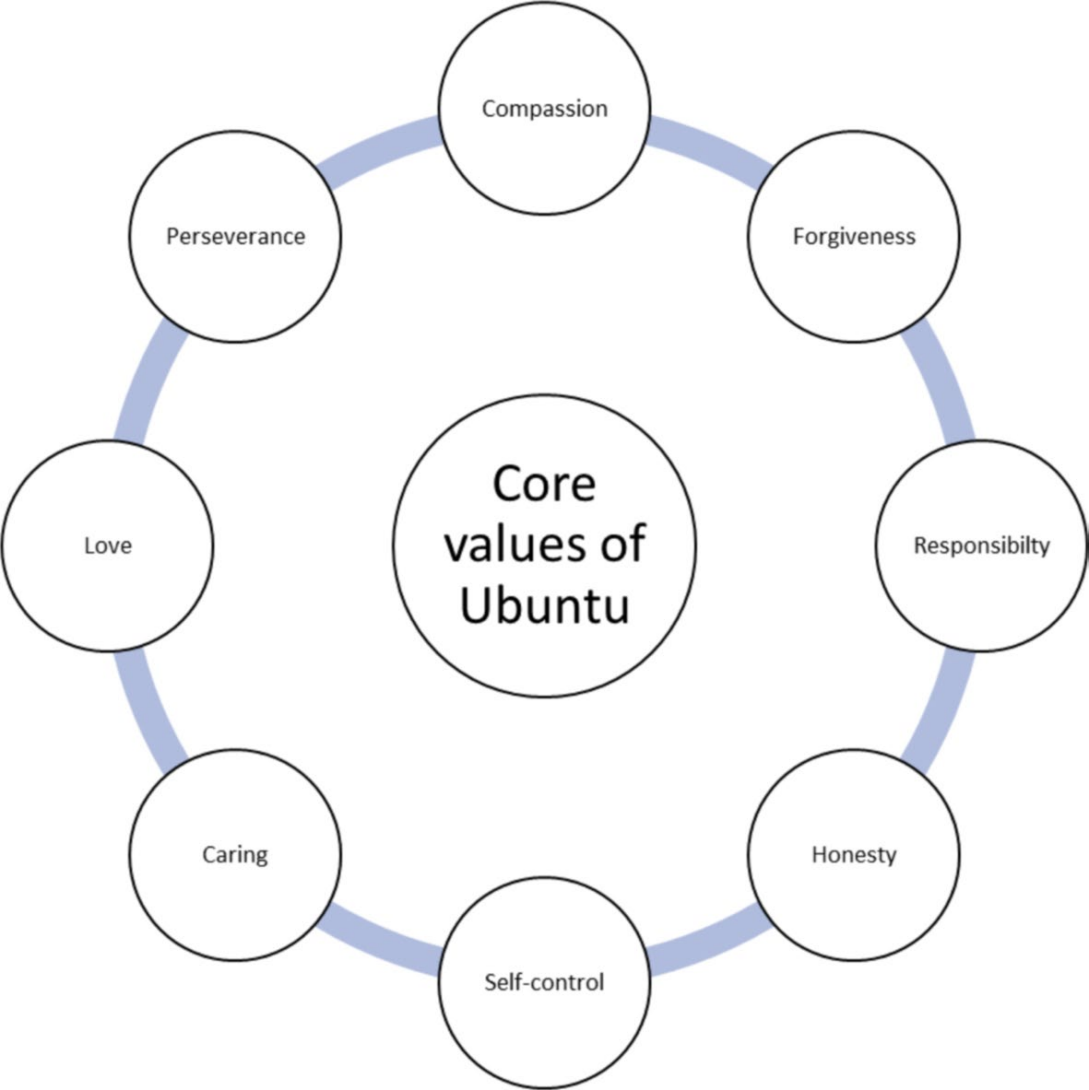
Eight principles of Ubuntu (Mangaroo-Pillay & Coetzee, 2022)

Ubuntu is an embodiment of humanism, which makes it ideal as a foundational overarching principle of professionalism from a Southern African perspective. The Ubuntu philosophy has eight overarching values that taken together guide the conduct of individuals in their interactions with others (Box 1 and Fig. 1).

**Box 1:** Mangaroo and Coetzee’s definition of Ubuntu

**“Compassion** (humaneness, human rights, humanity, spontaneity friendliness and helpfulness); **forgiveness** (understanding and consideration); **responsibility** (respect, obedience, giving unconditionally and sharing); **honest**y (good vs. bad and open-handedness; **self-control** (order, dignity, informality, redistribution and spirituality); **caring** (sympathy, appreciation and empathy); **love** (kindness, charity, tolerance and peace; and **perseverance** (strength, commitment and cohesion).” (Mangaroo-Pillay & Coetzee, 2022)
(Fig. 1)

**Fig. 2:**
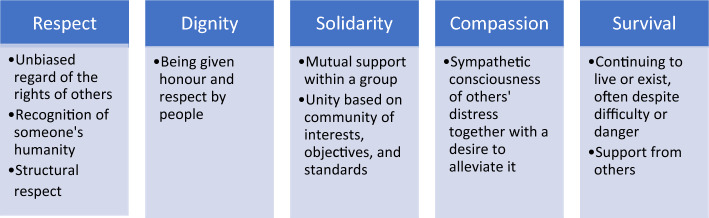
The definitions of respect, dignity, solidarity, compassion, and survival were used in the analysis section

Ubuntu philosophy uses metaphors instead of theories, for example, the hand in Mbigi’s Collective Finger Theory (Barac et al., 2021; Letseka, 2013; Ngubane & Makua, 2021). A thumb, although extraordinarily strong, is rendered useless without the rest of the fingers. As a collective, the fingers can form a fist that is hard to break. Mbigi developed five principles, one for each finger: solidarity/collectiveness, survival, compassion, respect and dignity to represent strength in unity and working together (Mbigi & Maree, 2005). Ubuntu embraces collectiveness and denounces the individualism celebrated by Western cultures; it could unite students and professionals from diverse cultural backgrounds and promote trustworthiness in the profession. If added to the professionalism discourse, Ubuntu might enrich the discourse resulting in other cultures recognizing and understanding what professionalism means for the Southern African-trained medical professionals. In this paper, we used various sources, including the Oxford and Merriam-Webster Dictionaries and Ubuntu literature to define the five values included in Mbigi’s metaphor (Letseka, 2012; Mangaliso, 2001; Mbigi & Maree, 2005; Molose et al., 2018) (Fig. 2).

### Research question

Guiding this study was the following research question, “*What are South African students’ experiences of professionalism during their journey towards becoming doctors and what does professionalism mean to them?”* This paper is written by the first author with input from the authorial team. Where the writing pertains to the first author’s voice, whose ontology is Ubuntu, the pronouns ‘I/me/my denotes her voice and experiences, in contrast, the pronouns ‘we/us/our’ denotes the voice of the full authorial team. We switch back and forth between voices to denote personal experiences from those of interpretation.

## Methodology and methods

Using students’ experiences in their clinical environment, my analysis, with the input of the authorial team, focuses on the relationship between Ubuntu and professionalism in the Southern African context. This study takes place within a broader study exploring medical students’ lived experiences during medical training between 2015 and 2020 at the University of the Witwatersrand in Johannesburg, South Africa (Mokhachane et al., 2022).

Regarding the three papers, the aim of the first paper was to describe and analyse the medical students’ and recent graduates’ worldviews and experiences centred around #FeesMustFall protests. (Mokhachane et al., 2022) The second paper was to explore graduates’ reflections and experiences of professionalism during three periods, i.e., during the fees must fall protests, the post-protests period and during their postgraduate training. (Mokhachane et al., 2023) The current analysis is to examine these SA students’ experiences of professionalism and what this construct means to them. In this paper, we aimed to go further in depth examine students’ experiences of professionalism and what it means to them. For this study, we selected eight students. Two previous articles used this same data set to explore their professional identity formation amidst protests and social upheaval, as well as the learners’ reflections on professionalism. In each of these articles, the thread of Ubuntu ran through them, (Mokhachane et al., 2022, 2023), and therefore this article focuses on this construct specifically.

### Methodology

The intention of this study was to understand the meaning of students’ encounters in the clinical space, so an interpretive phenomenological enquiry (Shaw & Anderson, 2021) was applied rather than a descriptive phenomenological approach (Mokhachane et al., 2022; Reiners, 2012).

### Sampling

Participants were all recruited in 2020 with students sampled from the final year class, while graduates were already working in various hospitals. These students were former leaders which I have interacted with in my role as a coordinator in the university but whom I was no longer supervising. Most of the participants were either in student leadership during the Fees Must Fall protests or activists. We chose the eight students because they specifically reflected on professionalism as a construct in the interviews. Choosing them for this paper was a way of finding out whether their differences impacted their experience of professionalism. Sample size was informed by information power (Malterud et al., 2016).

### Data collection

Semi-structured interviews were conducted with eight students in 2020 on their experiences in their medical training program (Table 1). We used an interview guide to probe the following areas: the journey to becoming a doctor, experiences of professionalism, and how #FeesMustFall protests impacted professional identity development (Mokhachane et al., 2022, 2023). The student participants comprised four women, one white and three black, and four men, two white and two black. The extracts from the participants’ comments are identified using BF for black female, BM for black male, WF for white female and WM for white male (Table 1). Three interviews were conducted in person, and five were mediated by an electronic platform, Microsoft Teams, due to pandemic lockdown restrictions.

**Table 1:** Summary of participants

Students at the time of study	Demography
S1BF	Black South African female
S2GWM (graduate entry)	White South African male
S3GWF (graduate entry)	White South African female
S4BM	Black African male
S5BF	Black South African female
S6WM	White South African male
S7BM	Black South African male
S8BF	Black South African female

### Data analysis

The interviews were transcribed verbatim and analyzed in MAXQDA 2020. Each transcript was analyzed as an individual case for the essence of its meaning and how participants’ experiences described influenced their being and becoming using an inductive approach (Matua & Van Der Wal, 2015; Reiners, 2012). Coding was conducted line by line or at the paragraph level (Azungah, 2018) to identify natural meaning units. Codes were grouped into categories and then into themes. The transcripts were read and coded by all team members to create a common codebook. Each interview was then profiled (the essence of each interview/case was discussed and described by the first author and further discussed and confirmed by the co-authors) and then compared for similarities and uniqueness. In-case and cross-case analyzes were applied, and ‘thematic connections’ were made across cases (Bazeley, 2009; Mokhachane et al., 2022; Wojnar & Swanson, 2007).

I (MM) did not impose an external theory or Ubuntu as a construct onto the data. This is because Ubuntu is the ontology through which the participants and I see the world, and in this study, I am making this visible to the reader. I did not use an a priori framework to apply to the data, but the students brought up Ubuntu, and I was able to see it because I knew how to recognize it in others’ words and experiences. As the primary author, I employed Ubuntu philosophy as an analytical lens concentrating on its eight core values and Mbigi’s metaphor, as mentioned in Figs. 1 and 2 (Mangaroo-Pillay & Coetzee, 2022; Mbigi & Maree, 2005). A deductive analysis then followed this using the framework in Fig. 2. For example, “I always give deference to those who are older” was a quote from one of the participants, which using the Ubuntu framework was coded as *respect*. This excerpt was coded as *respect* because, in South African culture, individuals are expected to obey elders, even if they are only a day older. The final level of analysis included grouping the participants’ perceptions of professionalism using the hand metaphor and then the eight values of Ubuntu were compared to definitions of professionalism outlined in the Physician’s Charter (Table 2).

### Positionality

I (MM) was trained in a white institution with a Western notion of professionalism, which sometimes conflicted with the Ubuntu values of my upbringing. The rest of the authorial team are educators, researchers, and administrators who work in the health professions education and have many international collaborations with individuals from different cultural backgrounds.

### Trustworthiness

To verify the trustworthiness of findings, when writing the graduates paper, the primary author asked participants to comment on the paper before submission, to ascertain whether they agree with the narration of their stories. However, this was not possible with the students as they had already left the institution by the time the transcriptions and the papers were written.

### Ethics

The study was approved by the University of Witwatersrand Human Research Ethics Committee on the 26th November 2019. (Clearance Certificate Number: M180864).

### Findings

**Fig. 3:**
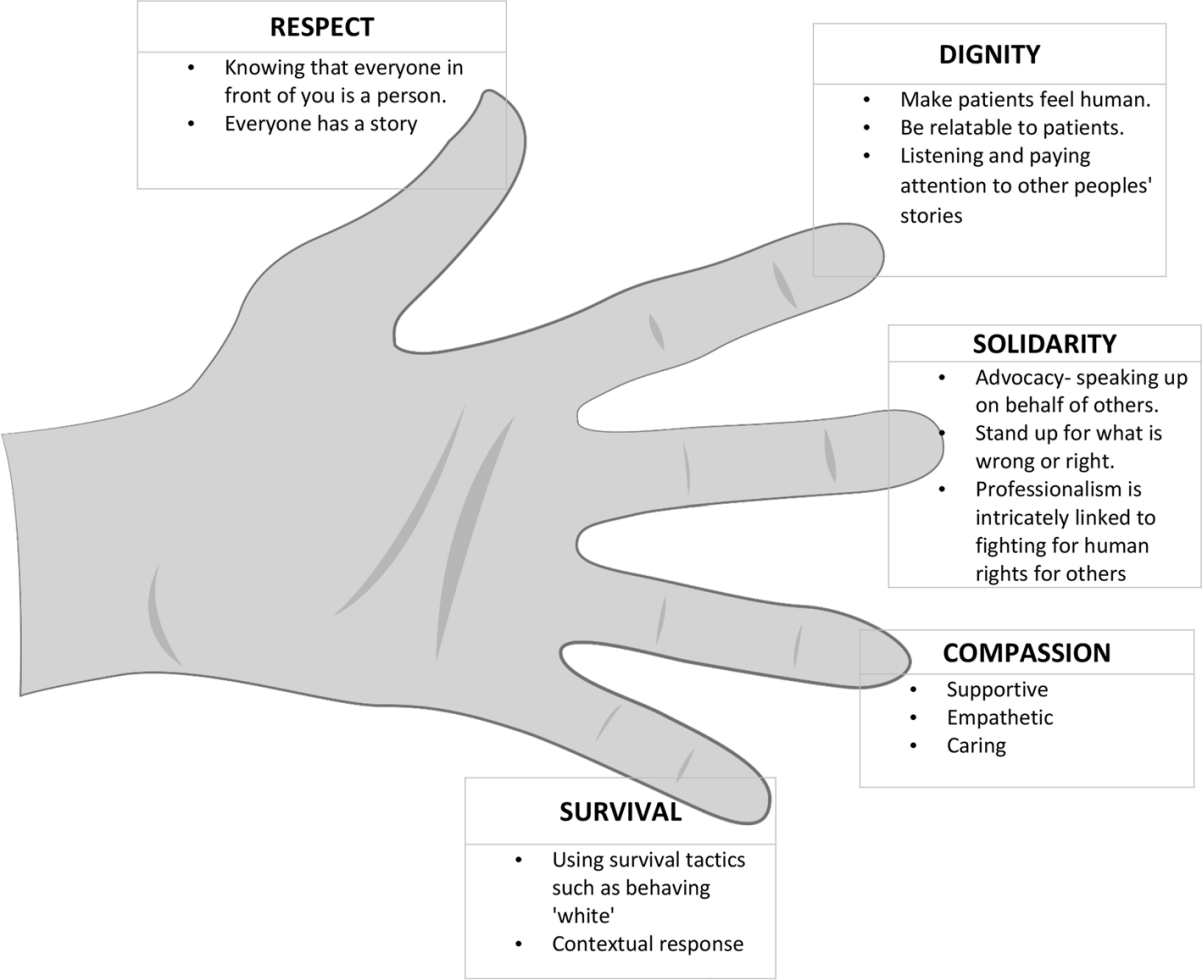
Participants view on professionalism

The results show that Ubuntu undergirds these eight South African medical students’ understanding of what it means to be a professional and how to enact professionalism in this context. To demonstrate the ways in which Ubuntu guides their understanding, we have organized the findings in the following ways. First, the participants’ experiences of professionalism and being socialized into being a physician using a narrative style and illustrated with quotes. Second, we use of Mbigi’s Collective Fingers Theory values, (Mbigi & Maree, 2005) namely respect, dignity, solidarity, compassion, and survival, as a means to present the data on what professionalism means to participants. (Mbigi & Maree, 2005) Examples from the data or quotes are included for clarity. This is followed by a diagram summarizing the second section, i.e., the participants’ experience of what professionalism meant to them, further categorized based on Mbigi’s Collective Fingers Theory (Fig. 3). Finally, Ubuntu was compared to the Physician’s Charter using the eight principles of Ubuntu (Table 2).

### Medical clerkship experiences

Participants found the clinical space hierarchical. The power dynamics silenced them to the extent of being unable to speak up on behalf of patients, where they observed unprofessional behavior from the clinicians they worked with. They were silenced in the name of “being professional” (S8BF) for “fear of victimization” (S8BF) and professionalism as a construct was used to override what matters. Participants witnessed some of their peers “adopting the hierarchical behavior” (S2GWM), which they found challenging, as this participant described,

*Medical profession is so hierarchical, and it is sickening to the stomach how hierarchical it is; I have experienced the most horrific…or witnessed, not personally experienced, the horrific, horrific hierarchical [behaviors] in medicine that are just so deeply ingrained that you can’t even say anything in certain areas. And just based on that one thing, your own view of professionalism can be shifted so much, you know, because when your future is [at] stake, for example, your seniors are the ones who are marking you and giving you your results and everything, you know you feel as though you just need to slot in, you just need to keep quiet, and you just need to get through until you are at a point in your life where you can start deciding what is professionalism and what is not* (S2GWM).

The hierarchy was silencing because participants feared victimization. It also led to a hidden curriculum breeding hierarchical behavior in the next generation of practitioners.

The participants insist that the current notion of professionalism “is old fashioned, generational” (S8BF), “judgmental” (S8BF; S7BM) and that it “ignores context” (S8BF). They spoke of the discrimination they faced in the clinical space, which included racism, structural racism, and favoritism (S4BM; S5BF; S7BM; S8BF), and those in positions of authority over them were dismissive of those who did not fit the norm, “[I was] frowned upon for speaking up and having tattoos” (S8BF).

According to participants, patients were on the receiving end of overworked clinicians and doctors’ unprofessional behavior. “Rape victims were negatively judged” (S7BM), “doctors being dismissive of patients” (S7BM), and “clinicians not following sterile precautions when performing procedures” (S2GWM). In multiple departments, patients were not treated with respect, and sometimes students felt as though patients were being sexually molested right in front of them judging by the clinician’s actions and what they said, for example, a male student saying:

*You know, it’s really sort of heart-breaking sometimes to see what some of the doctors do. I’ll share with you a few of these things but they’re quite sad and I still feel bad because I didn’t say anything, but I mean, I didn’t know what to do. But, for example, a doctor in [Y-department] [while conducting a vaginal examination on] a patient in front of everyone, while he’s PV-ing he’s saying, ‘This is nice and tight,’ exactly how I like it. He says that!* (S4BM).

This quote shows that the culture of medical education in South Africa does not consider Ubuntu. There were multiple examples where doctors were disinterested and disrespectful to patients and their peers. Without realizing it, doctors sent a negative hidden curriculum to the students about what it means to be a professional. Some students realized what type of doctors they did not want to be, while others adopted the behavior of the very clinicians who mistreated others.

The students raised negative and positive issues regarding their experiences of professionalism during their preclinical years, at the height of South Africa’s #FeesMustFall protests and throughout their clinical years. They saw patients treated dehumanizingly by those meant to assist them with their healing process. Sometimes patients’ treatment was based on judgement emanating from how they contracted their illness. For example, rape victims were made to feel as though it was their fault. Participants believed that every person has a story that needs to be heard with empathy and compassion. According to the participants, Ubuntu should be the foundation of how health professionals interact with patients, as discussed below.

In this next section, we present the participants’ data according to Mbigi’s Collective Fingers Theory of respect, dignity, solidarity, compassion, and survival.

### Collective Fingers theory

#### Respect

Respect was regarded as crucial, “not necessarily how you look but being open-minded and understanding of each other” (S8BF). Respect was also regarded as “knowing that everyone in front of you is a person” (S6WM) and that “everybody has a story” (S6WM), therefore “make them feel like human [beings]” (S6WM).

*We should treat each other with respect. We all come from different backgrounds, and we need to respect that. Respect where I come from as a Black woman; I hold myself to high standards. Respect is very important, not how you look but being open-minded and understanding of each other. Professionalism is outdated and not realistic in our setting. It is a privilege being in this profession- you hold these lives and make decisions for these lives. Be a human, not a title, not a career. It is a privilege that you are here. Put emphasis on respect, open-mindedness, and willingness to listen to other people’s stories* (S8BF).

Participants felt that respect has no color, age, gender, or social class. They posit that respecting everyone, irrespective of where they come from or how they look, was embracing their humanity.

#### Dignity

Participants propose that informality allows being “relatable to patients” (S8BF), which affords them dignity and yet carrying “oneself professionally” (S8BF) is still attainable. Participants were adamant that every person can be afforded dignity by listening to their stories before judging or treating them.

*The manner in which they treated patients, the manner in which they approach patients, the manner in which they…I can’t say harass, but demeaned the patients, undermined them. Not really understanding the reasons why they are here, why they have their illnesses, you know…some would even judge them for, you know…gynae, for example, they’d even suspect that the patient possibly went to go do an illegal abortion when it was not even the case. So those are the things, and it imprints on us as well because some people might accept that and see like that is normal. So, then you find that this very same student who becomes a doctor treats patients like that in future* (S7BM).

Patients were not treated as humans but as lesser beings.

#### Solidarity

Participants felt professionalism is “speaking or standing up on behalf of others” (S3WF) as they did during the protests. To them, “standing up for what is right or wrong” (S8BM; S2WM), advocacy was intricately connected to professionalism, and it was a “human rights issue” (S8BF).

*I think 2015 was just seeing student leadership as well as solidarity, that was the main positive, and the fact that we did get a zero percent increase, which allowed some students to come back into 2016. So, I think 2015 did have a fruitful result* (S1BF).

*I’d put it as advocacy because, for me, it’s like we work in such a messed-up healthcare system like it puts so much pressure on us and it overworks us because, like, we’re so little compared to the population we’re serving that we’re not able to being the necessary or the required kind of advocates for our patients that we can be already…you know what I mean? So, like, protesting to get the government…or to get whoever to clean up their act, the CEOs of the hospitals to clean up their act so that we can do our job better and become better advocates for our patients, who are unable to be advocates for themselves* (S8BF).

Supporting each other and being advocates for both their peers and patients was important to the participants.

#### Compassion

Maintaining self-control, where one has “to manage [one’s] anger for the sake of trying to help the patient” (S7BM), came first “no matter how angry one could be” (S7BM).

The participants perceived professionalism as “old fashioned and generational” (S8BF), while they also felt it as “silencing” (S7BM; S8BF), punitive and judgmental. They brought to the fore an example of a woman who was gang raped and managed to escape by jumping off a third floor of the building, fracturing her pelvis and legs. This “victim was judged” as if it were “her fault,” and due diligence was not afforded to her by the doctors who did “not use a rape kit” for medicolegal purposes (S7BM). According to the participants, “victim blaming” was a common phenomenon. There was a time when they were left to console a patient after an encounter with a clinician.

*And that everybody has a story, and that’s something I find super interesting in life. I mean, everybody has a story from where they started, where they’re now, what challenges they face, what things they overcame, what joys they’ve experienced in life. And it’s there, it’s like the only reason you can’t access it is because you’re not asking people or you’re not showing an interest in people’s lives* (S6WM).

Being cognizant that everyone has a story which might be heart-breaking, forces one to be compassionate to every person they meet, whether a patient or not. Doctors should make time to listen to patients’ stories. That might unearth the kindness underneath the facade doctors put up to hide their emotions.

#### Survival

For these participants, survival sometimes meant keeping quiet when seeing senior doctors behaving unprofessionally, as per the example above.

*So…ja…and there’s so many instances where you see how patients are being treated, from surgery to internal medicine, the care, you know. People would just dismiss patients without actually understanding why they are here. So those are the things I saw; the professionalism there was very poor, to be honest. Because there’s this hierarchy that, no, I’m the doctor, you’re the patient, and therefore we cannot interact as equals and try to understand why do you have this illness. How can I help you further? I’ve seen many a time, many a time. Even from gynae this year even certain doctors are very problematic. But then again, we’re like, you know what, we speak to certain like senior doctors, like no, we didn’t like one, two, three, with this particular doctor because they treated the patient in this manner. That’s how we go, but nothing could be done* (S7BM).

Survival does not always equate to speaking up and fighting, but sometimes in silence, one learns what to do or not do. They would return to the patient, comfort, and explain, clarify the situation, and answer some of their questions.

On reflecting, the participants said they had learnt what type of doctors they would like to be. For example, they would not want to be a doctor who disrespects patients and colleagues. They believed every person has a story, and they would endeavor to listen to their patients and colleagues alike. The participant’s experience of what professionalism meant to them was further categorized based on Mbigi’s collective finger theory (Fig. 3).

### Comparing the Physician’s Charter to Ubuntu

In the table below, we compared the three principles and the ten commitments of the Physician’s Charter to the eight values of Ubuntu displayed in Box 1 (Table 2). I did this because other people had tried to examine the Physicians Charter in South Africa. For example, Van Rooyen interrogated the applicability of the PC in the SA context, and her students did not entirely concur with the principles and responsibilities. They felt that the PC lacked humanistic aspects of professionalism (van Rooyen & Treadwell, 2007).

**Table 2:** Physician’s charter and Ubuntu. Modified from Nishigori et al. (2014)

Physicians charter	Ubuntu (respect overarching)							
Principle	Compassion	Forgiveness	Responsibility	Honesty	Self-control	Caring	Love	Perseverance
Primacy of patient’s welfare	**√**		√		√	√		√
Patient’s autonomy	√		√					
Social justice	√		√					√
*Commitment*								
Professional competence	√		√					
Honesty with patients	√		√	√				
Patient’s confidentiality	√		√		√			
Maintenance of appropriate relationships	√		√				√	
Improvement of quality of care	√		√					
Improvement of access to care	√		√					
A just distribution of finite resources	√		√			√		
Scientific knowledge	√		√					
Maintenance of trust by managing conflict of interest	√	√						
Professional responsibility	√		√	√	√	√	√	√

## Discussion

Scholars outside the Global North (North America, Europe, and Australia) have compared the Physician’s Charter with their context. Most of them compared this lofty, highly legitimate document, an encyclopedia of professionalism, with their context (Bano et al., 2021; Ho et al., 2014, 2016; Ibrahim et al., 2021; Jin, 2015; Jotkowitz & Glick, 2005; Nishigori et al., 2014; van Rooyen, 2004; van Rooyen & Treadwell, 2007). This phenomenon occurs because the Physician’s Charter has become a normative document, and everyone is interested in seeing how it compares to their specific context. In the published literature, it has been compared to various contexts, such as religious, cultural and Confucianism, among others (Al-Eraky et al., 2014; Ho et al., 2014; Nishigori et al., 2014).

However, when compared to Ubuntu, respect is found to be an overarching value and compassion includes all the principles and commitments stipulated in the Physician’s Charter (Blank, 2002). Responsibility is the next value that aligns with all three principles and all commitments but one. The Physician’s Charter’s professional responsibility commitment is aligned with all the Ubuntu values except forgiveness. Other values, such as forgiveness, honesty, self-control, caring, love, and perseverance, do not feature as much as compassion and responsibility do.

As participants discussed their experiences in becoming physicians, they alluded in their stories that attending physicians did not share the characteristics that embody Ubuntu, even though they were surrounded by patients and students who were born and socialized into Ubuntu households. South African medical education training including professionalism have imported the Global Northern way of delivery, for example what professionalism is (Mokhachane et al., 2022). The difference in cultural orientations could emanate from the physicians’ medical training, which may have espoused Western practices and behaviour. To these attending physicians that students referenced, some of which were Black and others white, there was only one right way of practicing medicine, namely the Western way. Hence the hierarchical, rigid, and silencing approach that lacks the humanity found in Ubuntu. I would postulate several other reasons for this behaviour. It could be the fruit of the hidden curriculum as they saw their seniors, particularly their role models, behaving that way and for them it has become the norm (Mokhachane et al., 2023). The academic public hospitals where these participants were trained are overcrowded, understaffed with an increasing lack of resources. This could also lead to burn out which might impact on how physicians interact with their patients.

This study demonstrates an Ubuntu specific way of expressing professionalism. We must therefore move away from the idea that professionalism is universally defined and instead see it as culturally and contextually bound. We urge the medical education community to look at professionalism from a specific cultural context as medical education training has become a global stage. Sensitization of international medical graduates deployed worldwide (particularly in Southern Africa) for training regarding cultural contexts is essential as their views of professionalism might conflict with an Ubuntu-infused understanding. Therefore, Western medical education or medicine globally should consider the person’s cultural background, just as Ubuntu does, before passing judgement on their actions around professionalism.

In the spirit of what Nishigori started, moving away from the ‘them and us’ dialogue, we decided to add Ubuntu to the discussion, where the Physician’s Charter was compared to Ubuntu (Table 2) (Nishigori et al., 2014). Doing this does not necessarily say they are the same, but it might confirm that context does speak to the universal Physician’s Charter appropriately for the local context. There are similarities between Ubuntu and the Physicians Charter. However, the depth of humanness in Ubuntu goes beyond what is mentioned in the Charter. This is in keeping with the study conducted by Van Rooyen, where students stressed the lack of humanity in the Physicians Charter (van Rooyen, 2004). Whereas the Charter talks about ‘commitments’, which is an appropriate term, it has a connotation of being distant and militaristic (Blank, 2002; Blank et al., 2003). No matter how noble the idea of a universalist idea of professionalism might be, it lacks Ubuntu, that sense of humanness that says the doctor is not merely doing this as a duty, but also because they feel a sense of deep-seated caring for the humanity of someone else (Waghid & Smeyers, 2012). Talking about the language used in the Physicians’ Charter matters because in the Ubuntu philosophy, words matter, and they should match the deeds portraying a sense of oneness (Masango, 2006).

In Ubuntu, the saying ‘I am because we are’ emphasizes the importance of community. The participants spoke about the importance of knowing the story of the person they met. This is in keeping with the African epistemology, which starts with “community and moves to the individual” (Hailey, 2008). Therefore, the doctor needs to inquire more about the person’s community and how those individuals fit into that community. This was reinforced to the first author recently by people she met on her travels in the southern part of South Africa. When these people introduced themselves to her, they would mention where they came from before their names. This confirms the critical role communities play in their lives. This is in keeping with other ways of knowing and being, as displayed in Confucianism, Bushido, Arabic, Islam, and Judaism. Confucianism has a strong cultural influence and social history, highlighting the importance of context, where morality and integrity are paramount as guiding principles (Ho et al., 2014; Wong, 2020). Al-Rumayyan found commonalities between the Chinese, Japanese and Arabic cultural frameworks (Al-Rumayyan et al., 2017). As we have entered a global stage, we all need to be sensitive to others’ backgrounds which might impact how they see professionalism.

This study is, however, not without limitations. Firstly, the sample was small, and it is possible that with a larger sample, the findings could have been different. Secondly, those who agreed to participate might have been people who are passionate about the well-being of others hence their alignment to Ubuntu philosophy.

In conclusion, this study adds an African voice to the professionalism discourse, while also showing that there are African elements that could be aligned to the Physician’s Charter to bring about a greater sense of humanity.
